# Management of Ascites in Patients with Cirrhosis: An Update

**DOI:** 10.3390/jcm10225226

**Published:** 2021-11-10

**Authors:** Giacomo Zaccherini, Manuel Tufoni, Giulia Iannone, Paolo Caraceni

**Affiliations:** 1 Department of Medical and Surgical Sciences, University of Bologna, 40138 Bologna, Italy; giacomo.zaccherini@unibo.it (G.Z.); giulia.iannone@studio.unibo.it (G.I.); 2 IRCCS AOU di Bologna—Policlinico di S. Orsola, 40138 Bologna, Italy; manuel.tufoni@aosp.bo.it; 3 Center for Biomedical Applied Research, University of Bologna, 40126 Bologna, Italy

**Keywords:** decompensated cirrhosis, portal hypertension, effective hypovolemia, anti-mineralocorticoids, loop diuretics, vaptans, TIPS, human albumin

## Abstract

Ascites represents a critical event in the natural history of liver cirrhosis. From a prognostic perspective, its occurrence marks the transition from the compensated to the decompensated stage of the disease, leading to an abrupt worsening of patients’ life expectancy. Moreover, ascites heralds a turbulent clinical course, characterized by acute events and further complications, frequent hospitalizations, and eventually death. The pathophysiology of ascites classically relies on hemodynamic mechanisms, with effective hypovolemia as the pivotal event. Recent discoveries, however, integrated this hypothesis, proposing systemic inflammation and immune system dysregulation as key mechanisms. The mainstays of ascites treatment are represented by anti-mineralocorticoids and loop diuretics, and large volume paracentesis. When ascites reaches the stage of refractoriness, however, diuretics administration should be cautious due to the high risk of adverse events, and patients should be treated with periodic execution of paracentesis or with the placement of a trans-jugular intra-hepatic portosystemic shunt (TIPS). TIPS reduces portal hypertension, eases ascites control, and potentially modify the clinical course of the disease. Further studies are required to expand its indications and improve the management of complications. Long-term human albumin administration has been studied in two RCTs, with contradictory results, and remains a debated issue worldwide, despite a potential effectiveness both in ascites control and long-term survival. Other treatments (vaptans, vasoconstrictors, or implantable drainage systems) present some promising aspects but cannot be currently recommended outside clinical protocols or a case-by-case evaluation.

## 1. Introduction

The development of ascites is the most frequent decompensation event in patients with liver cirrhosis. Five to ten percent of patients with compensated cirrhosis per year develop ascites, an event that represents a cornerstone in the natural history of the disease, so that it has become accustomed considering it the hallmark of the transition to decompensated cirrhosis [1]. Indeed, it often marks the border between a stable and a turbulent clinical course, burdened with acute events of decompensation, including acute-on-chronic liver failure (ACLF), bacterial infections, and frequent hospitalizations, thus determining a dramatic worsening in quality of life and prognosis [2]. Consequently, 5-year survival drops from ~80% in compensated patients to ~30% after ascites onset, and the overall median survival is around two years [1].

Such unfavorable prognosis can be explained—at least partially—by considering that ascites development results from the concurrence of multiple and interrelated pathogenetic mechanisms, involving splanchnic and systemic hemodynamics, along with liver and extrahepatic organs dysfunction (mainly kidney and heart) [3]. Therefore, the onset of ascites presupposes that such abnormalities have reached a critical threshold.

In this context, an appropriate treatment of ascites is a crucial goal in managing patients with cirrhosis [4]. Preventing ascites onset and controlling its evolution means offering the patients a better quality of life, reducing the incidence of acute decompensations and emergency hospitalizations, and improving survival, thus also leading to a more appropriate long-term allocation of healthcare resources [4].

The present review, after a brief recall of the main pathogenetic mechanisms underlying decompensation and ascites formation in patients with cirrhosis, will discuss the currently available approaches for ascites management, along with some emerging perspectives and areas for future research.

## 2. Pathophysiology of Ascites and Decompensation

The classical pathophysiological paradigm of ascites formation in patients with liver cirrhosis relies on the so-called peripheral arterial vasodilatation hypothesis [5]. The primary event is the progressive disruption of the normal structure of the liver that leads to portal hypertension as a result of the increased intrahepatic vascular resistance and sinusoidal pressure. In turn, portal hypertension favors the production of endogenous vasodilating substances, such as nitric oxide (NO), endocannabinoids, and carbon monoxide (CO), that exert their action on systemic vascular resistances, mainly affecting the splanchnic arteriolar bed which becomes abnormally dilated. Because of this dysregulated splanchnic vasodilation, effective hypovolemia develops. Effective hypovolemia is the crucial event in the pathogenetic cascade of decompensation in patients with liver cirrhosis, as it causes the activation of neuro-humoral systems able to promote vasoconstriction and renal retention of sodium and water, such as the renin–angiotensin–aldosterone (RAA) axis, the sympathetic nervous system (SNS), arginine–vasopressin (ADH), thus producing a compensatory increase in cardiac output. As the disease progresses and these mechanisms are sustained over time, the exhaustion of left ventricular function and the development of cirrhotic cardiomyopathy could lead to an impairment of cardiac output and a further decrease of effective volemia, thus leading to peripheral hypoperfusion and contributing to multi-organ failure [5].

This pathophysiological interpretation, however, does not fully explain all the clinical manifestations of decompensated cirrhosis, with the subsequent need to integrate it with new fundamental discoveries. In recent years, indeed, newly available evidence led to consider systemic inflammation and immune system activation as major drivers of organ impairment and failure in decompensated cirrhosis [3]. The key event is a dysregulated activation of the immune system from two major drivers: first, portal hypertension increases intestinal mucosal permeability and favors the translocation from gut lumen of pathogen-associated molecular patterns (PAMPs), such as bacterial products, lipopolysaccharide (LPS), and bacterial DNA [6], and, second, the chronic liver damage with hepatocyte necrosis releases circulating damage-associated molecular patterns (DAMPs), intracellular components released by dying or damaged host cells [7]. The resulting increased production of cytokines and other proinflammatory molecules, reactive oxygen species (ROS) and vasodilating substances can cause peripheral organ damage and failure via tissue hypoperfusion, immune-mediated tissue damage, and mitochondrial dysfunction [3]. The detailed discussion of these complex mechanisms, however, falls beyond the scope of this review.

## 3. Diagnosis of Ascites

Cirrhosis and portal hypertension are the main causes of ascites, accounting for about 80% of cases in Western countries, but many other etiologies, such as malignancies, congestive heart failure, nephrotic syndrome, or tuberculosis, may be responsible of ascites formation [8]. Previous patient’s history, physical examination, laboratory tests, abdominal ultrasound and diagnostic paracentesis are therefore recommended in all patients with new onset ascites [4].

A serum–ascites albumin gradient (SAAG) of 1.1 mg/dL or above is suggestive of the presence of portal hypertension and helps to discriminate the underlying condition when the causative disease is unclear [9]. Therefore, in case of paracentesis, ascitic total protein and albumin concentration should be measured. Moreover, neutrophil count and ascitic fluid culture should be routinely performed to exclude spontaneous bacterial peritonitis (SBP), a severe and potentially life-threatening complication in patients with decompensated cirrhosis [4,10]. To notice, an ascitic total protein concentration below 1.5 mg/dL may identify patients at high risk for SBP development, so that a long-term antibiotic prophylaxis could be considered [11,12]. Other ascitic fluid analysis (e.g., cytology or culture for mycobacteria) could be performed, depending on clinical suspicion [4].

As regards a quantitative classification, ascites could be graded as mild, moderate, or severe (grade 1 to 3) according to the total amount of fluid in the abdomen [8].

## 4. Management of Uncomplicated Ascites

With the term “uncomplicated ascites” it is generally defined any ascites that is not refractory, not infected nor associated with renal failure (i.e., hepatorenal syndrome) [8]. Grade 1 (or mild) ascites does not generally require a specific treatment since only few data on its long-term evolution and prognosis are available, nor clear evidence on the effects of therapies on its natural history [4,13]. In patients developing grade 2 (moderate) ascites, a specific treatment should be initiated [4].

### 4.1. Dietary Salt Restriction

As a positive sodium balance is a major determinant of ascites accumulation, the reduction of dietary salt intake and the increase of renal sodium excretion are the two cornerstones of moderate ascites management. Dietary salt restriction should be suggested with caution and carefully supervised: although low-sodium diets can induce or support ascites resolution in a portion of patients [14], an extreme or exaggerated salt restriction could even favor hyponatremia and renal failure [15], along with a worsening in nutritional status, due to a reduced calories intake and impaired food palatability [16]. Therefore, international guidelines currently recommend a moderate salt restriction (medium intake of 80–120 mmol/day), mainly to avoid excessive intake [4].

### 4.2. Diuretic Therapy

The initiation of a diuretic therapy has the goal to induce natriuresis and consequently a negative sodium balance. From a mechanistic point of view, diuretics can be considered symptomatic treatments, not clearly affecting the general course of the disease, since they act downstream in the pathophysiological cascade. As already reported, the main pathogenetic mechanism of renal sodium retention in patients with decompensated cirrhosis and preserved renal perfusion is secondary hyperaldosteronism [17]. Therefore, the mainstays of ascites treatment so far are the anti-mineralocorticoid drugs, such as spironolactone, canrenone or potassium-canrenoate, at the initial dose of 100 mg/day, up to a maximum of 400 mg/day [4]. These drugs block the aldosterone pathway in the distal convoluted tubule through a slow action (involving cytosolic and nuclear receptors), so that the natriuretic effect begins after 72 h from the first dose and dose changes should be managed accordingly. If patients could not be treated with anti-mineralocorticoids due to intolerance or severe adverse effects, amiloride could be used. Amiloride acts on aldosterone pathway in the renal collecting duct, but it is less effective than spironolactone [18].

As a second step, in non-responder patients (defined as subjects presenting a weight loss of less than 2 kg/week or side effects such as hyperkalemia) or in patients with long lasting ascites, a combination therapy should be considered, adding loop diuretics (furosemide at a starting dose of 25–40 mg, and up to 160 mg in 25–40 mg steps) to anti-mineralocorticoids [4]. Indeed, as portal hypertension progresses, proximal tubule sodium reabsorption becomes relatively prevalent due to the activation of RAAS and SNS and reduced renal perfusion [19]. Therefore, combining loop diuretics to anti-mineralocorticoids has been demonstrated to be more effective in controlling ascites than anti-mineralocorticoids alone, also preventing serum potassium alterations [20]. In patients showing an inadequate response to furosemide, torasemide can be considered, as it showed a more effective natriuresis in one randomized trial [21].

The target of therapy in patients with moderate to severe ascites is a weight loss of maximum 0.5 kg/day in patients without peripheral edema, and of maximum 1 kg/day in patient with peripheral edema, to avoid the development of renal impairment and adverse effects, such as hyponatremia [22]. In parallel with the effective mobilization of ascites (i.e., its consistent reduction until resolution), diuretic therapy dosage should be gradually reduced to the minimal effective dose [4].

In case of high dose diuretic therapy, especially at the beginning, patients should be frequently monitored to notice adverse effect (Table 1). The most common side effects of furosemide are hyponatremia and hypokalemia, while anti-mineralocorticoids can lead to hyperkalemia and painful gynecomastia. Moreover, diuretic-induced rapid reduction of extracellular volume or electrolyte imbalance can favor the occurrence of other severe complications such as overt HE, acute kidney injury (AKI) until renal failure, and muscle cramps. According to the severity of side effects, dose reduction or even temporary interruption of diuretic therapy could be necessary [4] (Table 1).

### 4.3. Therapeutic Paracentesis

In patients developing grade 3 (severe/tense) ascites, large volume paracentesis (LVP) represents the treatment of choice due to its efficacy and low rate of complications [4]. The procedure is associated with a very low risk of bleeding, even in patients with altered international normalized ratio (INR > 1.5) and platelet count < 50,000/microl [23]. LVP should be avoided in patients with disseminated intravascular coagulation; moreover, no evidence supports the routine use of fresh frozen plasma or pooled platelets before LVP execution in case of mild or moderate coagulopathy [4]. Although not routinely recommended by international guidelines, the use of bedside ultrasound guidance can reduce the incidence of adverse events, particularly in settings where LVPs are performed by non-physician healthcare providers [24].

After drainage of large volumes of ascitic fluid (especially > 5 L), plasma volume expansion is recommended to avoid paracentesis-induced circulatory dysfunction (PICD), a severe syndrome due to the acute worsening of effective hypovolemia and the consequent increase in plasma renin activity, leading to renal failure, severe hyponatremia, hepatic encephalopathy (HE), and eventually death [25]. Human albumin, at the recommended dose of 8 g per liter of tapped ascites, has been demonstrated to be the plasma expander of choice and should be administered to all patients undergoing LVP [4,25]. Other plasma expanders (such as dextran-70, polygeline, or saline solution) show similar efficacy in preventing PICD compared to albumin only in case of small volume paracentesis (<5 L) [26,27]. Drainage of less than 5 L could require human albumin administration in case of concomitant of acute-on-chronic liver failure (ACLF) or in patients at high risk of renal failure development [24,28].

### 4.4. Referral for Liver Transplantation

All patients with cirrhosis and grade 2 or 3 ascites should be considered for liver transplantation (LT) [4,24]. The presence of hyponatremia, a reduced renal sodium excretion and glomerular filtration rate, and hypotension are all predictors of mortality in these patients [29]. Therefore, a major issue in this setting is the appropriate prioritization for organ allocation, since nor Child–Pugh score, nor MELD/MELD-Na score fully reflects the potentially poor prognosis of patients with ascites [30,31]. Indeed, in case of an excessive time on waitlist for LT, decompensated patients could develop further complications and severe acute events (mainly infections) leading to a significant clinical deterioration. The development of innovative and specific prognostic tools for patients with ascites is a major objective for future research.

## 5. Management of Refractory Ascites

Refractory ascites (RA) is generally defined as “ascites that cannot be mobilized or the early recurrence of which (i.e., after LVP) cannot be satisfactorily prevented by medical treatment” [8], both for a progressive lack of response to diuretic therapy (diuretic-resistant RA) and for the development of diuretics-related complications (diuretic-intractable RA). To notice, the diagnostic criteria for RA should be evaluated in clinically stable patients, without recent acute complications. Recurrent ascites, which is defined as ascites that recurs at least three times within 1 year despite dietary sodium restriction and adequate diuretic dosage, could be a forerunner of RA, although its natural history and prognostic significance are only partially known [8,13].

Besides challenges in the therapeutic management of patients, RA also dramatically worsen patients’ prognosis, reducing the median survival to about six months. Therefore, a prompt evaluation for LT or the immediate referral to a transplant center are strongly recommended for any patients with RA [4,32]. In this regard, as already reported, a major problem is the appropriate patient prioritization on the waitlist. Indeed, the main liver function parameters could often be only moderately altered, so that the main prognostic scores (Child-Pugh and MELD/MELD-Na) do not fully reflect patient urgency. Some proposals have been made to refine patients’ priority and improve organ allocation. This is the case, for example, of the Italian Score for Organ allocation (ISO), that introduce some “MELD exceptions” and provide additional points to patients, based on specific complications that heavily affect prognosis and ease further complications [33].

Periodic execution of LVPs is generally agreed to be the treatment of choice—both effective and safe—for patients with RA [4,32]. Plasma volume expansion with albumin (8 g/L of tapped ascites) should always follow any LVP to prevent PICD [34]. A lower dose of albumin (4 g/L of tapped ascites) has been proposed in patients undergoing paracentesis of less than 5 L of ascites drained [35]. According to the major guidelines, in these cases the use of albumin could be administered on a case-by-case basis (e.g., patients at risk of renal impairment or failure) [4,32].

As regards diuretic therapy, it should be modulated or withdrawn due the high risk of diuretic-related adverse effects, such as worsening in glomerular filtration rate and electrolytes disturbances [8]. Instead, non-selective beta-blockers (NSBBs) could be administered unless severe hypotension, hyponatremia or renal failure develop, as clarified in the BAVENO VI consensus [36]; to note, carvedilol is not recommended at this stage [4,37].

## 6. Trans-Jugular Intra-Hepatic Portosystemic Shunt

Currently recommended therapeutic strategies for patients with decompensated cirrhosis and ascites act downstream on the complex pathophysiological cascade leading from portal hypertension to ascites development. Indeed, the mainstays of ascites treatment, anti-mineralocorticoids, loop diuretics and LVPs, can be considered symptomatic treatments from a mechanistic point of view. The trans-jugular intra-hepatic portosystemic shunt (TIPS), conversely, addresses portal hypertension, a key upstream event in the pathophysiology of decompensated cirrhosis.

TIPS consists of creating an artificial shunt between portal and hepatic vein, thus decreasing portal hypertension. Currently, the main clinical settings for the use of TIPS are the management of variceal bleeding and the control of refractory ascites [38]. TIPS leads to an increase in cardiac output and a decrease in systemic vascular resistance. Therefore, it causes an improvement in effective hypovolemia and renal perfusion, thus inducing natriuresis [39]. In clinical practice, however, the main contentious points on TIPS placement remain the identification of target patients and appropriate timing for its use. The main side effect of TIPS is the occurrence or worsening of HE [40]; moreover, major possible complications are related to dysfunction due to stent stenosis or thrombosis [41]. The introduction of polytetrafluoroethylene (PTFE)-covered stent, currently the standard of care, instead of bare stent grafts has been shown to significantly reduce these risks [42,43]. Absolute and relative contraindications to TIPS placement are reported in Table 2.

In patients with decompensated cirrhosis and ascites, seven RCTs [44,45,46,47,48,49,50] compared TIPS to LVPs plus albumin, the standard of care for patients with RA, showing a superior efficacy in controlling ascites in all of them. However, beyond a beneficial effect on ascites, the trials showed slightly different results. Indeed, TIPS positively affected survival when performed using PTFE-covered stents [50] and in patients with a less advanced disease [49] or recurrent’/recidivant’ ascites not fulfilling criteria for refractory ascites [45,48,50]. At the same time, TIPS placement requires great caution and a careful selection of target patients, because of the high risk of adverse events, such as hepatic encephalopathy, liver failure, and cardiac dysfunction [38]. The use of smaller diameter stents (6–8 mm of diameter instead of standard 10 mm) seems promising for the expansion of TIPS indications, since they showed a similar efficacy with a lower incidence of adverse events, likely by preventing excessive shunting [42,43].

In summary, addressing a key pathophysiological mechanism as portal hypertension, TIPS eases ascites control and shows a great potential in increasing patients’ survival. However, two factors currently limit its widespread use in clinical practice: First, available studies evaluated TIPS efficacy in patients with portal hypertensive bleeding or difficult-to-treat/refractory ascites; its impact in patients with clinically significant portal hypertension but without these complications has never been assessed. Second, the occurrence of severe TIPS-related complications often prevents its placement in patients with RA refractory ascites. The use of small diameter covered stent may help in overcoming these limitations.

## 7. Long-Term Human Albumin Administration

Albumin use in patients with cirrhosis is currently recommended for the treatment or prevention of conditions characterized by an acute worsening of effective volemia: its well-established indications are the prevention of paracentesis-induced circulatory dysfunction (PICD), of renal dysfunction induced by SBP, and the diagnosis and treatments of HRS in association with vasoconstrictors [4,32]. Indeed, with its oncotic properties, albumin can counteract effective hypovolemia, a central event in cirrhosis pathophysiology. At the same time, albumin molecule exerts several functions not related to its oncotic power (the so called non-oncotic properties), including antioxidant activities, binding with many endogenous and exogenous substances, modulation of immune response and inflammation, restoration of endothelial integrity, and cardiac function [51]. These pleiotropic effects make it a multitarget agent in a mechanistic perspective, thus supporting a potential role in modifying the long-term clinical course of decompensated cirrhosis.

Recently, the ANSWER trial [52] showed for the first time that long-term albumin administration, on top of a standard diuretic therapy, could be a novel therapeutic approach for patients with cirrhosis and grade 2–3 uncomplicated ascites. Indeed, albumin administration obtained a 38% reduction in 18-month mortality hazard ratio, eased the management of ascites (with a 50% reduction in the need for LVPs and RA diagnosis) and reduced the incidence of major complications of cirrhosis [52]. Following the positive results of the ANSWER trial, a single-center non-randomized trial showed that long-term human albumin administration could improve 24-month survival in patients with RA [53]. The results of the ANSWER trial have been challenged by the MACHT trial, that did not obtain differences between the two arms, both in survival and in the incidence of complications of cirrhosis [54]. However, instead of simply advise against long-term albumin use, the careful comparison of the two studies can provide essential information for its appropriate use [55]. The different results could be at least partially explained by differences in disease severity of patients enrolled (slightly less severe in the ANSWER trial), and dosage and duration of albumin treatment (higher and longer, respectively, in the ANSWER trial) (Table 3).

One of the most important findings of the ANSWER trial was the increase of serum albumin concentration (by approximately 0.6–0.8 g/dL), leading to the normalization of albuminemia (up to close 4 g/dL) in treated patients [52]. This result was further explored in a post hoc analysis [56] that showed two interesting findings: first, the best 18-month survival probability (greater than 90%) was obtained by patients reaching an on-treatment serum albumin concentration of at least 4 g/dL (not only a normalization above 3.5 g/dL); second, baseline MELD score and serum albumin value independently predicted the achievement of this threshold. Consequently, it could be assumed that patients with severe hypoalbuminemia and high MELD score could require greater amounts of albumin to obtain long-term beneficial effects. Last, the serum albumin threshold of 4 g/dL was not arbitrarily assumed, as such a concentration represents the normal serum albumin level in healthy individuals in their eighth or even ninth decade [57].

In summary, growing evidence support long-term albumin use in patients with decompensated cirrhosis and ascites, showing a potential role in modifying the natural history of the disease, beyond the treatment of ascites or other specific complications. Further studies are needed to better characterize target subgroups, who could benefit the most from this innovative approach, and to establish different dosages and schedules of albumin administration, ideally tending to an individualization of treatment. However, the effectiveness of chronic albumin administration is still a debated issue worldwide, and the major international guidelines do not recommend long-term albumin as an established treatments [4,32]. The only exception, so far, is represented by the recently released Italian clinical practice guidelines [58], that include albumin among the medical treatment options for decompensated patients with ascites.

## 8. Other Proposed Treatments for Ascites

### 8.1. Vaptans

Vaptans antagonize vasopressin by blocking V2 receptors in the renal collecting ducts, thus inducing diuresis without excretion of electrolytes [59]. Their use in managing ascites with hyponatremia is controversial. Indeed, these drugs improved serum sodium concentration in patients with hyponatremia and eased ascites control, according to two metanalyses [60,61], although no benefits were demonstrated on cirrhosis complications or mortality. However, a small single-center real-life study did not show the effectiveness of vaptans in patients with severe hyponatremia [62], perhaps due to reduced response to the treatment related to renal impairment, common in advanced cirrhosis. So far, the available studies did not demonstrate a clear survival benefit of vaptans in patients with ascites. Interestingly, a non-randomized single-center clinical trial showed an improvement in survival in a subgroup of patients, treated with Tolvaptan and a low-dose of furosemide [63]. Although interesting, these results need further confirmations to be generalized.

The European Medicines Agency (EMA) approved the administration of vaptans only for the syndrome of inappropriate antidiuretic hormone secretion (SIADH). On the other hand, the Food and Drug Administration (FDA) also included heart failure and cirrhosis, until 2013. In 2013, indeed, cases of severe hepatic damage occurred in patients with autosomal dominant polycystic renal disease treated with vaptans in a trial [64], so that FDA currently do not recommend the use of vaptans in patients with liver disease.

In conclusion, no evidence currently supports the routine use of vaptans in patients with cirrhosis and ascites. Moreover, according to some clinical practice guidelines, vaptans should only be administered for a short period of time in hospital setting with strict electrolytes monitoring, due to the risks of a rapid sodium correction [4,65].

### 8.2. Midodrine and Clonidine

Splanchnic vasodilation plays a major role in the development and maintenance of effective hypovolemia, a key step in the pathophysiology of ascites in cirrhosis. Therefore, the use of vasopressor such as midodrine, an alfa-adrenergic agonist, could theoretically help in the management of ascites. Indeed, in non-azotemic patients with ascites, midodrine showed an increase in mean arterial pressure and in renal sodium excretion, with a decrease in plasma renin activity and aldosterone [66]. Two small RCTs showed benefits of midodrine in the control of ascites [67,68], although larger studies are needed to confirm these findings. A possible alternative is clonidine, an alfa-2-adrenergic agonist which blocks RAA and SNS activity and, in association with diuretics, may enhance diuretic response [69,70]. Based on the currently available evidence, however, the use of midodrine or clonidine in patients with cirrhosis and ascites could not be recommended and should be considered only on a case-by-case basis.

### 8.3. Automated Low-Flow Ascites Pump (Alfapump)

The Alfapump is a subcutaneously implanted battery-powered programmable pump connected to catheters that move ascites from the peritoneal cavity to the bladder, from which it is eliminated with urine. It could be considered, in experienced centers, for patients with RA and contraindications to TIPS placement. The available evidence shows that Alfapump can reduce the need for LVPs and improve patients’ quality of life and nutritional status [71,72]. However, important side effects have been reported and deserve consideration. First, device-related infective complications are relatively frequent (mainly SBPs and urinary tract infections) [73]; the routine use of antibiotic prophylaxis reduced their occurrence, but long-term antibiotic administration remains a debated issue in patients with decompensated cirrhosis. Second, renal impairment or failure develop in a proportion of patients [73,74], probably as a form of PICD due to the continuous ascites tapping without albumin use. Intermittent albumin administration has been proposed but its efficacy (as well as its dose and timing) needs to be demonstrated in clinical trials. Moreover, no survival benefit of Alfapump has been showed so far [73]. In conclusion the routine use of Alfapump is currently not an established option in patients with cirrhosis and refractory ascites.

## 9. Conclusions

Currently recommended treatments for ascites are based on symptomatic measures, aiming to excrete the excess of water and sodium by the kidney (with diuretics) or directly drain ascites from the abdomen (with LVPs). Alternative approaches, like vasoconstrictors (i.e., midodrine) or automated drainage systems (i.e., alfapump) present some promising aspects but did not show clear and undoubted beneficial effects, so far.

Future research should focus on pathophysiological treatments, able to treat or prevent ascites in a wider context, ideally modifying the long-term clinical course of the disease, thus improving survival and quality of life for patients [75]. Such measures could derive from a better knowledge—and extended use—of currently available treatment (e.g., TIPS and albumin administration), from the repurposing or repositioning of existing drugs [76], or even from the development of innovative approaches or molecules.

## Figures and Tables

**Table 1:** Complications and adverse events related to diuretic therapy and recommendations on diuretics management according to major international guidelines [4].

Adverse Event/Complication	Recommendations
Renal failure or acute kidney injury	Discontinuation (or at least reduction) of diuretic therapy
Overt hepatic encephalopathy	
Severe hyponatremia (<125 mmol/L)	
Incapacitating muscle cramps	
Severe hyperkalemia (>6 mmol/L)	Anti-mineralocorticoids withdrawal
Severe hypokalemia (<3 mmol/L)	Loop diuretics withdrawal

**Table 2:** Contraindications to TIPS placement.

Absolute Contraindications	Relative Contraindications
Very advanced disease (Child-Pugh > 13)	Hepatic tumors (especially if centrally located)
Overt or recurrent hepatic encephalopathy	Obstruction of all hepatic veins
Congestive heart failure	History of episodic hepatic encephalopathy
Severe tricuspid regurgitation	Portal vein thrombosis
Severe pulmonary hypertension (mean pulmonary pressure > 45 mmHg)	Severe thrombocytopenia (<20,000/microL)
Polycystic liver disease	Mild/moderate pulmonary hypertension
Active systemic infection or sepsis	
Unrelieved biliary obstruction	

**Table 3:** Comparison of the two available RCTs on long-term albumin use for decompensated cirrhosis and ascites [52,54].

Feature of the Study	Answer Trial [51]	Macht Trial [53]
Study design	Randomized Open label	Randomized Placebo-controlled
Number of patients	431 (218 HA/213 SMT)	173 (87 HA/86 SMT)
Baseline MELD score	12/13	17/18
Albumin dose	40 g weekly (with a loading dose of 40 g twice a week for the first 2 weeks)	40 g every 2 weeks (+midodrine)
Duration of treatment	17.6 (8.0–18.0) months ^§^	63 days ^‡^
Effects on albumin concentration	Increase in SA level (0.6–0.8 g/dL) in about 4 weeks	No changes in SA levels
Outcomes of the interventional arm	Reduction of mortality and complications of cirrhosis	No effect on mortality or complications

^§^ duration of follow-up in the treated group according to reverse Kaplan–Meier method; ^‡^ median duration of follow up in the treated group. HA, human albumin; SMT, standard medical treatment; MELD, model for end-stage liver disease; SA, serum albumin.

## Data Availability

No new data were created or analyzed in this study. Data sharing is not applicable to this article.
